# Physical Properties of Five Brands of K-Files

**DOI:** 10.7508/iej.2016.02.008

**Published:** 2016-03-20

**Authors:** Arash Izadi, Arash Shahravan, Hoda Shabani Nejad

**Affiliations:** a * Laboratory Sciences Research Center, Golestan University of Medical Sciences, Gorgan, Iran; *; b * Endodontology Research Center, Kerman University of Medical Sciences, Kerman, Iran; *; c *Kerman University of Medical Sciences, Kerman, Iran*

**Keywords:** Corrosion, Debris, Physical Properties, Scanning Electron Microscopy

## Abstract

**Introduction:**

Endodontic K-files are major tools for cleaning and shaping of the root canal systems. As there are various K-files available in Iranian market, the physical properties of the five available brands were investigated to assist the clinician when selecting suitable endodontic K-files according to the intended application.

**Materials and Methods:**

Physical properties (including debris creation, machinery defect and corrosion) of the selected K-files were investigated by a scanning electron microscope (SEM) under ×250 magnification. For evaluating the flutes number, a stereomicroscope was used with ×40 magnification.

**Results:**

Maximum and minimum debris and corrosion were observed in the Larmrose and Perfect K-files, respectively. Dentsply showed the least machinery defects. Other brands had intermediary properties. In addition, Larmrose K-files showed the maximum flutes number compared to the other brands.

**Conclusion:**

According to the results, none of the K-files had the ideal properties. More studies regarding the physical properties of the K-files and their clinical efficacy are suggested.

## Introduction

The specifications of any hand file such as shape of the tip, number of flutes, the symmetry of the tip and the space between the flutes have a significant effect on root cleaning [1]. Literature show the need for further research on standardization of endodontic instruments [2]. Sotokawa *et al. *[3] studied the causes for instrument failure to develop clinical measures to prevent occurrence of such fracture. Keate *et al*. [4] studied the tip angulations and four visual characteristics denoting manufacturing quality of endodontic files using photo-micrographs. Stenman and Spangberg [5] examined the topology of nine different brands of Hedstrom files, seven brands of K-files and four brands of special files using a computerized measuring microscope. Miserendino *et al. *[6] investigated the cutting efficiency of endodontic instruments. Measurements were made of the angles and lengths, and the topology of the tips on seven endodontic instruments and the results were examined under microscope. They observed significant differences between various tip designs. According to Felt *et al. *[7], the tip of the file showed a greater cutting efficiency than the flutes. In addition, an increase in the distance between the flutes led to an increase in the volume of excavated material from canal walls. They evaluated the cutting efficiency of four brands and three sizes of endodontic files and reamers. Filho and Esberard evaluated three types of as-received and used files morphometrically using a stereomicroscope. According to the results, most unused stainless-steel files suffered from manufacturing defects [8]. Dearing *et al. *[9] evaluated the physical properties of two hand files including torque at failure, angular deflection at failure, flexibility, and consistency of diameter at 3 mm from the cutting tip. 

Craig and Peyton [10] studied the physical properties of stainless steel and carbon steel files. They found that stainless steel instruments hold considerable promise as endodontic instruments. Some recent works focused on the specifications of the rotary instruments [11-15]. Serene *et al. *[16] measured variations in same-size endodontic files with a three-dimensional gauge. Their results showed that about 74% of variations stemmed from the design of files. Stenman *et al.*[17] studied the machining efficiency of endodontic K-files and Hedstrom files. They suggested that in order to evaluate the machining and cutting of root canal files and also to provide the endodontist with better information about instruments, similar standardized procedures must be introduced.

Some recent studies focused on the impact of physical properties of K-files on their cutting efficiency [18-20]. Working characteristics should not be the only criteria in selection of endodontic instruments, but the reactivity of the metal in the working environment should also be considered. An appropriately selected file will improve the speed and efficiency of treatment.

There are variable brands of files in Iranian market. Although this diversity provides the clinicians with many options, it may be confusing if their specifications are not thoroughly studied. The aim of the present study is to evaluate the physical properties of five brands of K-files available in Iranian market.

**Table 1:** The number of corroded files and the location of corroded zone along the different brands of k-files

**Perfect**		**Larmrose**		**Thomas**		**Mani**		**Dentsply**			
No	Yes	No	Yes	No	Yes	No	Yes	No	Yes		
5	1	2	4	4	2	5	1	5	1	**Tip**	**#** **15 **
5	1	1	5	4	2	5	1	5	1	**Middle**	
4	2	2	4	4	3	6	0	5	1	**End**	
4	2	0	6	2	4	4	2	4	2	**Total**	
6	3	3	3	5	1	5	1	4	2	**Tip**	**#** **20 **
4	2	2	4	6	0	6	0	2	4	**Middle**	
5	1	1	5	6	0	6	0	2	4	**End**	
4	2	1	5	5	1	5	1	1	5	**Total**	
6	0	4	2	5	1	4	2	2	4	**Tip**	**#** **25 **
6	0	4	2	3	3	3	3	2	4	**Middle**	
6	0	1	5	2	4	2	4	1	4	**End**	
6	0	1	5	1	5	2	4	0	6	**Total**	
6	0	5	1	6	0	3	3	3	3	**Tip**	**#** **30 **
4	2	4	2	6	0	3	3	5	1	**Middle**	
4	2	4	2	6	0	3	3	4	2	**End**	
4	2	2	4	6	0	1	5	3	3	**Total**	
23	1	14	10	20	4	17	7	14	10	**Tip**	**Total**
19	5	11	13	19	5	17	7	14	10	**Middle**	
19	5	8	16	17	7	17	7	12	11	**End**	
18	6 (25%)	4	20 (83%)	14	10 (41%)	12	12 (50%)	8	16 (66%)	**Overall**	

**Table 2:** The location of debris for K-files of different sizes

**Perfect**		**Larmrose**		**Thomas**		**Mani**		**Dentsply**			
No	Yes	No	Yes	No	Yes	No	Yes	No	Yes		
2	4	4	2	3	3	3	3	3	3	**Tip**	#15
3	3	2	4	5	1	4	2	5	1	**Middle**	
3	3	3	3	2	4	2	4	5	1	**End**	
1	5	0	6	1	5	0	6	2	4	**Total**	
2	4	0	0	3	3	1	5	1	5	**Tip**	# 20
4	2	0	0	2	4	1	5	2	4	**Middle**	
5	1	0	0	2	4	1	5	4	2	**End**	
2	4	0	0	1	5	1	5	1	5	**Total**	
5	1	1	5	1	5	2	4	2	4	**Tip**	#25
6	0	0	0	3	3	3	3	2	4	**Middle**	
6	0	0	0	4	2	2	4	4	2	**End**	
5	1	0	0	1	5	1	5	1	5	**Total**	
4	2	2	4	1	5	3	3	2	4	**Tip**	#30
4	2	3	3	5	1	5	1	6	0	**Middle**	
5	1	1	5	5	1	4	2	6	0	**End**	
4	2	0	6	1	5	3	3	2	4	**Total**	
13	11	7	17	8	16	9	15	8	16	**Tip**	Total
17	7	5	19	15	9	13	11	15	9	**Middle**	
19	5	4	20	13	11	9	15	19	5	**End**	
12	12 (50%)	4	20 (83%)	4	20 (83%)	5	19 (79%)	6	18 (75%)	**Overall**	

**Figure1:**
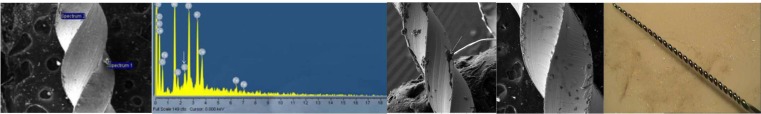
*A) *SEM micrograph of a file. Note the cavities on the surface of the file (×250 magnification); *B)* EDX spectrum of the cavities, *C)* A #25 Mani instrument under ×250 magnification. The white arrow shows debris in the middle of the file; *D)* A #25 Larmrose instrument under ×250 magnification. Machinery defects in the middle of the file, *E)* The flutes of a #25 Mani instrument under ×40 magnification

**Figure 3:**
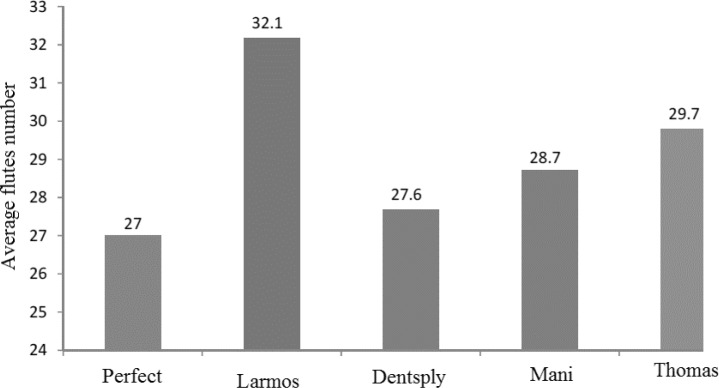
The number of flutes in the studied files

## Materials and Methods

Five brands of K-files including Dentsply (Dentsply Maillefer, Ballaigues, Switzerland), Mani (Mani, Tochigi, Japan), Thomas (French Dental Products, Société FFDM-PNEUMAT, Département Dentaire Thomas, Bourges Cedex, France), Larmrose (Taizhu, China, Beijing) and Perfect (Shenzhen, China, Guangdong) were selected. From each brand, six files from each size (#15 to 30) were selected (*n*=24).

***Image preparation***

In this study, stereomicroscope micrographs were taken to obtain the number of flutes in the K-file brands. A scanning electronic microscope (SEM), CamScan MV2300 (CamScan, Cambridge, UK**)** was applied to investigate other physical properties including corrosion, machinery defect and debris. For this purpose, the rapid-setting cyanoacrylate glue was first applied on the SEM disks and then the main part of the files were separated from the plastic handle and fixed on the disks. One side was fixed on the disk and the flutes were filled with glue. Then the SEM micrographs were taken from the other side of the files free of the cyanoacrylate glue. SEM micrographs were taken from the tip, middle and the end regions of the files under ×250 magnification and 1 kVp voltage. The micrographs were saved as JPEG images. The results were included in tables by two blind observers. A third person evaluated the data in the case of disagreement between the two observers.

***Characterization***

***Corrosion***

The existence of any cavity on the surface of the files was considered as a sign of corrosion [21]. Figure 1A shows the SEM micrograph of a file. Some small cavities are obvious on the outer surface of the file. These corroded points were assessed by energy dispersive x-ray microanalysis (EDX). The presence of sulfur in the EDX spectrum confirms the occurrence of corrosion [22]. For instance, Figure 1B shows the results of EDX analysis for the cavities in Figure 1A. The presence of sulfur in the spectrum confirms occurrence of some corrosion in the file.

***Debris***

SEM micrographs (×250 magnification) were taken to detect the machining debris in tip, middle and end of the file (Figure 1C). 

***Machinery defect***

Any visible defect in the file structure is considered as a machinery defect. This includes discontinuity of flutes, flute curvature, metal flushes or any other observable structural defect after opening the package of the files [23]. In Figure 1D, machinery defects are clearly seen in the middle of the file.

***Flutes number***

The number of shiny points in the stereomicroscope micrograph (under ×40 magnification) is defined as flutes number. For instance, the number of flutes in the file shown in Figure 1E is 27.

## Results

***Corrosion***

The stereomicroscope and SEM micrographs of the K-files of different brands were analyzed and the following results were obtained. According to Table 1, the highest corrosion rate was observed in Larmrose files; 20 out of 24 files showed corrosion in the tip, middle and end of the file. Dentsply took the second place in terms of corrosion severity and 16 out of 24 files showed corrosion to some extent. The lowest corrosion was observed in Perfect files; only 6 out of 24 specimens were corroded. Table 2 lists the location of corrosion in the files. As can be seen, the tip and end of Larmrose and Dentsply files had the highest corrosion rate while the Larmrose showed the lowest corrosion in the tip and end of the Perfect files.

***Debris***

SEM micrographs were used to examine the presence of debris in the K-files. Table 2 shows debris observed in different areas of the files (tip, middle and end). According to the results, the most amount of debris was observed in Larmrose and Thomas files. In addition, a significant diversity was detected in the existence of debris in the middle part of the studied files. In other words, 19 out of 24 Larmrose files (79.2%) showed debris in the middle part showing a significant difference with the other brands. The least debris was observed in the Perfect files.

***Machinery defects***

Table 3 lists the number of machinery defects and their locations in the studied files. As can be seen, the lowest machinery defects were seen in Dentsply files (one defect in tip, one in the end and overall two defects) whereas Larmrose (18 defects), Mani (18 defects) and Thomas (16 defects) had the highest machinery defects, respectively.

***Flutes number***

The average flutes number in the studied files is shown in Figure 3. The Larmrose files showed the highest flutes number, while the lowest number of flutes was found in Perfect files. A significant difference was observed between these two brands while the other brands showed no significant difference.

## Discussion

In this study, five brands of available hand K-files marketed in Iran were selected after consulting with some endodontists. The number of flutes was obtained from the stereomicroscope micrographs taken from the files with a proper magnification. SEM micrographs with acceptable accuracy, were used to study the presence of debris, corrosion and machinery defects in the hand files.

Corrosion is a defect that may cause cavity and finally separation in Nickel-Titanium and stainless steel instruments. This phenomenon is crucial when corrosive materials such as hypochlorite are used. According to Cormier *et al. *[24], the occurrence of corrosion in a file is independent of the file brand and cost and even a cheap brand may have low corrosion rate in comparison with an expensive one, as approved by the present study. Formerly, Parirokh *et al*. [25] proved that almost all endodontic files possess metallic and non-metallic debris before autoclaving.

The existence of debris in a file can be a sign of contamination that should be considered carefully. Some amounts of debris was observed in a large number of files immediately after unpacking, it is necessary to pay more attention to the infection control procedures and sterilization before operation.

Newman *et al. *[26] demonstrated that the higher number of flutes in a file, can improve its cutting performance. In the present study, the highest flutes number was observed in Larmrose files. Files of the Perfect brand showed the lowest flutes number. It can be assumed that Larmrose files can remove tissues more effectively than Perfect files, but it remains matter of discussion for future studies in this field. 

**Table3:** The number and location of machinery defects in the studied files

**Perfect**		**Larmrose**		**Thomas**		**Mani**		**Dentsply**			
**No**	**Yes**	**No**	**Yes**	**No**	**Yes**	**No**	**Yes**	**No**	**Yes**		
5	1	3	3	3	3	1	5	6	0	**Tip**	**#** **15 **
5	1	1	5	4	2	3	3	6	0	**Middle**	
5	1	3	3	4	2	1	5	5	1	**End**	
3	3	1	5	3	3	1	5	5	1	**Total**	
6	0	3	3	6	0	5	1	6	0	**Tip**	**#** **20 **
5	1	2	4	4	2	4	2	6	0	**Middle**	
6	0	1	5	6	0	3	3	6	0	**End**	
5	1	1	5	4	2	2	4	6	0	**Total**	
5	1	4	2	5	1	2	4	6	0	**Tip**	**#** **25 **
4	2	3	3	2	4	3	3	6	0	**Middle**	
6	0	3	3	2	4	1	5	6	0	**End**	
3	3	1	5	1	5	1	5	6	0	**Total**	
6	0	6	0	1	5	3	3	5	1	**Tip**	**#** **30 **
6	0	4	2	0	6	3	3	6	0	**Middle**	
6	0	4	2	0	6	2	4	6	0	**End**	
6	0	3	3	0	6	2	4	5	1	**Total**	
22	2	16	8	15	9	11	13	23	1	**Tip**	**Total**
20	4	10	14	10	14	13	11	24	0	**Middle**	
23	1	11	13	12	12	7	17	23	1	**End**	
17	7 (29%)	6	18 (75%)	8	16 (66%)	6	18 (75%)	22	2 (8%)	**Overall**	

Any defects in a file that has occurred during the manufacturing process are called machinery defects. These defects may accelerate corrosion propagation and ultimately cause the breaking of a file during practice [27]. Anderson *et al*. [28] suggested electropolishing as a post processing treatment after manufacturing to eliminate the machinery defects in the files [28]. The manufacturers are highly recommended to apply electropolishing after file preparation and before packing. According to the present study, Larmrose files showed the highest machinery defects while the lowest number of machinery defects was observed in Perfect and Dentsply files. Despite the modern manufacturing technology of Perfect files, they are not much expensive than the other brands of K-files studied.

## Conclusion

According to the results, none of the brands of K-files investigated demonstrated ideal properties for endodontic treatment. Perfect files exhibited better properties in terms of lower corrosion rate and debris. The lowest number of machinery defects was observed in Dentsply files. Larmrose files showed the lowest number of flutes compared to other brands. 
